# Label-free microfluidic enrichment of cancer cells from non-cancer cells in ascites

**DOI:** 10.1038/s41598-021-96862-y

**Published:** 2021-09-09

**Authors:** Nicholas E. Stone, Abhishek Raj, Katherine M. Young, Adam P. DeLuca, Fatima Ezahra Chrit, Budd A. Tucker, Alexander Alexeev, John McDonald, Benedict B. Benigno, Todd Sulchek

**Affiliations:** 1 The George W. Woodruff School of Mechanical Engineering, Georgia Institute of Technology, Atlanta, GA 30332 USA; 2 Department of Mechanical Engineering, Indian Institute of Technology Patna, Bihar, 801103 India; 3 Wallace H. Coulter Department of Biomedical Engineering, Georgia Institute of Technology, 313 Ferst Drive, Atlanta, GA 30332-0535 USA; 4 Department of Ophthalmology and Visual Science, Carver College of Medicine, Institute for Vision Research, University of Iowa, Iowa City, IA 52242 USA; 5 School of Biology, Georgia Institute of Technology, 313 Ferst Drive, Atlanta, GA 30332-0405 USA; 6 Ovarian Cancer Institute, Atlanta, GA USA

**Keywords:** Cancer, Cell biology, Biomarkers, Translational research

## Abstract

The isolation of a patient's metastatic cancer cells is the first, enabling step toward treatment of that patient using modern personalized medicine techniques. Whereas traditional standard-of-care approaches select treatments for cancer patients based on the histological classification of cancerous tissue at the time of diagnosis, personalized medicine techniques leverage molecular and functional analysis of a patient's own cancer cells to select treatments with the highest likelihood of being effective. Unfortunately, the pure populations of cancer cells required for these analyses can be difficult to acquire, given that metastatic cancer cells typically reside in fluid containing many different cell populations. Detection and analyses of cancer cells therefore require separation from these contaminating cells. Conventional cell sorting approaches such as Fluorescence Activated Cell Sorting or Magnetic Activated Cell Sorting rely on the presence of distinct surface markers on cells of interest which may not be known nor exist for cancer applications. In this work, we present a microfluidic platform capable of label-free enrichment of tumor cells from the ascites fluid of ovarian cancer patients. This approach sorts cells based on differences in biomechanical properties, and therefore does not require any labeling or other pre-sort interference with the cells. The method is also useful in the cases when specific surface markers do not exist for cells of interest. In model ovarian cancer cell lines, the method was used to separate invasive subtypes from less invasive subtypes with an enrichment of ~ sixfold. In ascites specimens from ovarian cancer patients, we found the enrichment protocol resulted in an improved purity of P53 mutant cells indicative of the presence of ovarian cancer cells. We believe that this technology could enable the application of personalized medicine based on analysis of liquid biopsy patient specimens, such as ascites from ovarian cancer patients, for quick evaluation of metastatic disease progression and determination of patient-specific treatment.

## Introduction

Metastasis is the primary cause of cancer-related death. The American Cancer Society has predicted 21,750 new diagnoses of ovarian cancer and 13,940 deaths due to ovarian cancer in 2020^1^. Metastasis plays a large role in ovarian cancer related mortality, given that 75% of ovarian cancer patients already have metastatic disease at the time of diagnosis, resulting in a five year survival rate of only 48.6%^1,2^.

Detecting and analyzing metastasizing cancer cells in locations of the body in which the cancer cells are a minority population remains a challenge. A consequence of the inability to identify and isolate rare metastatic cells for molecular characterization and drug testing is the inability to optimize chemotherapies; this contributes to the lack of progress in addressing metastatic cancers. A variety of techniques have been developed to enrich cancer cells, usually requiring conjugation of antibodies to surface antigens, which may not be specific to the cells of interest. For example, a magnetic bead capture and isolation immunoassay^3,4^, while sensitive to ovarian cancer cells, is expensive to implement and requires the use of antibodies against specific surface markers that may not be present on cancer cells or may also exist on healthy cells. Similar limitations exist with fluorescence activated cell sorting (FACS). While an adherence assay has been developed to enrich ovarian cancer cells^5^, which does not rely on the use of antibody conjugation and sorting, this method requires 2–3 days for cells to be adhered, washed, and cultured.

To improve the accuracy of downstream analyses of metastatic cells, there is a need for label-free and high-throughput methods for enriching cancer cells within fluids, which includes effusions, ascites, lymph, and blood. The benefits of obtaining more highly purified cancer cell samples include increased sensitivity to gene expression diagnostics. Higher purity samples will then result in accelerated cancer biology research and improved treatments by clinicians through more accurate and sensitive outcomes of analytical techniques. Enriching cancer cells will also enable molecular readout methods, for example ELISA, PCR, and FISH, to enhance scientific discovery, such as determining whether prognostic markers of primary tumors differ from cells in effusions^6^. Ovarian cancer is a particularly important pathology to apply enrichment techniques, considering the general poor quality of existing biomarkers^7–9^. For example, one marker used to assess malignancy in ovarian cancer is TGM2, but unfortunately the resulting protein TG2 is expressed in a wide variety of tissues and detectible in all organs^10^.

Detection of circulating tumor cells can rely on differences in tumor cell biomechanical properties, especially cell size. Moreover, other cell biomechanical properties have shown use as biomarkers for enriching cancer cells from non-cancer cells. Using several different experimental techniques, abnormalities in the biophysical properties of tumor cells have been widely studied in primary and cultured cells^11–13^ with specific examples including prostate cancer^14^, bladder cancer^15^, breast cancer^16–18^, esophageal cancer^19^, thyroid cancer^20^, oral cancer^21^, ovarian cancer^22^, pancreatic cancer^23^, and leukemia^24,25^. The molecular mechanisms for the change in cell stiffness are likely a result of remodeling of cytoskeletal pathways^26^ and nuclear composition^27^. The mechanical properties of exfoliated cancer cells have been shown to undergo drastic alterations compared to and distinct from healthy counterparts. Cross et al. have quantified breast cancer cell stiffness, by a parameter called Young’s modulus, and showed a correlation of stiffness with cell malignancy^28^. In their work, the stiffness of metastatic cancer cells taken from the pleural fluids of patients with breast cancer is more than 70% softer, with a standard deviation over five times narrower, than benign reactive mesothelial cells. Similar results were obtained using different methodologies by Guck et al. in breast epithelial cells^16^. These results suggest biomechanical analysis can distinguish cancerous cells from noncancerous cells, even if their morphologies are similar^29^. In prior work studying the mechanical properties of ovarian cancer cell lines, we have used atomic force microscopy (AFM) to show that more invasive ovarian cancer cells are softer than less invasive cells^26^ and nonmalignant epithelial cells, indicating that cell stiffness may be a useful biomarker for use in diagnosis of ovarian cancer and isolation of metastatic cells.

While several microfluidic approaches have been described for the high frequency measurement of cell stiffness^16,24,30^, the number of methods available for sorting of mechanically distinct cell types is fairly restricted. Several approaches have been developed to sort cells based on size^31,32^. Two approaches for sorting cells by size include hydrodynamic focusing and ferrohydrodynamic cell separation. In hydrodynamic focusing, cells are pumped at high speed down microfluidic channels. A balance of forces due to drag and wall induced lift then dictate that cells occupy equilibrium positions in the channel which are a function of their size. This approach, while high throughput and simple suffers from low sensitivity given that changes in cell size only cause a moderate change in equilibrium position. Another size-based sorting method is ferrohydrodynamic cell separation. When using this approach, cells are immersed in a ferrofluid and pumped through a channel which is placed in a magnetic field. The interactions of this ferrofluid with the magnetic field induces a buoyancy force on the cells proportional to their volume. Stiffness-based sorting methods primarily rely on one of two approaches: (1) confined geometries (i.e. pillars), which slow the flow of stiff cells^33^ or (2) inertial focusing, which cause stiff cells to translate laterally in the channel with respect to soft cells due to nonlinear effects in channel flow^34,35^. A limitation of the pillar approach is that samples are processed relatively slowly with low throughput. A limitation of the inertial focusing approach is that the sensitivity to cell stiffness is small, as soft and stiff cells displace only a fraction of a cell diameter. The limited sensitivity requires more precise flow control, making it difficult to obtain multiple, biophysically distinct outputs and improved fractionation of heterogeneous cells. To address the need for a high-throughput label-free enrichment strategy for malignant ovarian cancer cells, we demonstrate the optimization and use of a microfluidic device for the isolation of malignant cells from primary ascites samples. The device design is similar to those used previously to isolate retinal cells and stem cells^36,37^. The microfluidic device itself is biologically inert and sorts cells based on their mechanical and physical properties into biologically meaningful fractions. These fractions can be tailored based upon modification of the device parameters and flow conditions. To facilitate the optimization of this platform for different sorting applications we have developed a computational model that couples the resistance to cell deformation, determined from cell size and measured Young’s modulus, as well as hydrodynamical forces of the flow (3D flow trajectories) to model the trajectories of the cell under the ridge. The results are described in more detail in^38^.

## Methods

### Device design and fabrication

The microfluidic sorting devices were fabricated using standard photolithography and replica molding process. Silicon masters fabricated using photolithography were prepared to mold a polydimethylsiloxane (PDMS) chip replicating the microchannel design. The PDMS chip was prepared with inlet and outlet holes formed with a biopsy punch and bonded onto a glass slide using oxygen-plasma bonding (Harrick Plasma, USA). The fabricated devices were passivated using 1% BSA solution by incubating for one hour at 37 °C to reduce the non-specific adhesion of cells with the channel surface. The number of ridges and the angle was chosen to be 14 and 30° respectively, based upon an optimization of hydrodynamic and elastic forces^38^. The gap size was chosen to be 9 μm to provide sufficient compression for sorting while not causing excessive cell deflection in stiffer populations.

### Cell line preparation and sorting

Ovarian cancer cell lines were used to optimize the microfluidics processing. OVCAR-3 and HEY-A8 were originally provided by Dr. G. Mills (MD Anderson Cancer Center, Houston, TX) and cultured in the laboratory using RPMI-1640 media with 10% FBS and 1% penicillin-streptomyocin. Once the cells were 70% confluent, they were washed with phosphate buffered saline (PBS) (without calcium and magnesium) and dyed using following procedure. OVCAR-3 and HEY-A8 cells were dyed with Cell Tracker Deep Red and Cell Tracker Green CMFDA respectively as per the protocol provided by the manufacturer (ThermoFisher). Then, the cells were trypsinized and mixed with the flow buffer (35% Percoll, 1% BSA, 1% EDTA and 0.006% Tween in PBS[-,-]) at a concentration of approximately 5 × 10^6^ cells/mL and infused into the device at a flow rate determined to result in the best separation of cell samples. Each device inlet was connected to a syringe pump (PHD-2000, Harvard Apparatus) using plastic tubing, Luer lock adapters and blunt Luer lock needles. The cell sample was then infused at a flow rate of 15 μL/min. Flow buffer was infused into the left and the right sheath inlets at 25 and 10 μL/min respectively to position the cell flow stream optimally off-center in the channel. The sorted cells were collected at the outlets using pipette tips and counted using flow cytometry.

### Trajectory analysis

The trajectories of the ovarian cancer cell types were recorded using high-speed optical microscopy (Vision Research Phantom v9.0). Videos from each cell type were analyzed using ImageJ to identify where individual cells came into contact with each ridge. Video pixels were converted to microns and video tilt between the primary flow direction and video orientation were corrected using a custom R program. This program (https://github.com/nstone8/Manual-Tracking) was used to convert the locations at which the cell impacted each ridge into cumulative deflection per ridge data to characterize trajectory of each cell type.

### Ascites sorting for NGS sequencing

Primary ascites specimens were obtained from two patients from Northside Hospital (Atlanta, Georgia) (Fig. 1.1). All patients provided written, informed consent for this study, which was approved by the Central Institutional Review Board of the Georgia Institute of Technology (protocol number H16135) and adhered to the tenets set forth in the Declaration of Helsinki. These specimens were processed with a cell strainer (pluriSelect) to remove solid tissue and large cell aggregates greater than 20 um in size (Fig. 1.2). Cells were collected via centrifugation (Fig. 1.3) and resuspended in flow buffer containing 35% Percoll, 1% BSA, 1% EDTA and 0.006% Tween in PBS[-,-] and pumped through our device at a total flow rate of 50 ul/min (Fig. 1.4). Cells were then collected from the outlets and frozen at − 80 °C for sequencing analysis. Genomic DNA was then isolated from the sorted cells using a Nucleospin Tissue kit (Machery-Nagel). Next Generation Sequencing (NGS) libraries for sequencing TP53 were prepared using an Accel-Amplicon Comprehensive TP-53 kit (Swift Biosciences) (Fig. 1.5). Samples were then barcoded using unique adapter sequences, pooled and sequenced on a single micro flow cell of an Illumina MiSeq (Fig. 1.6). A known disease-causing mutation (c.455dup) was observed in the sequencing data of both patients along with wild type reads.

**Figure 1:**
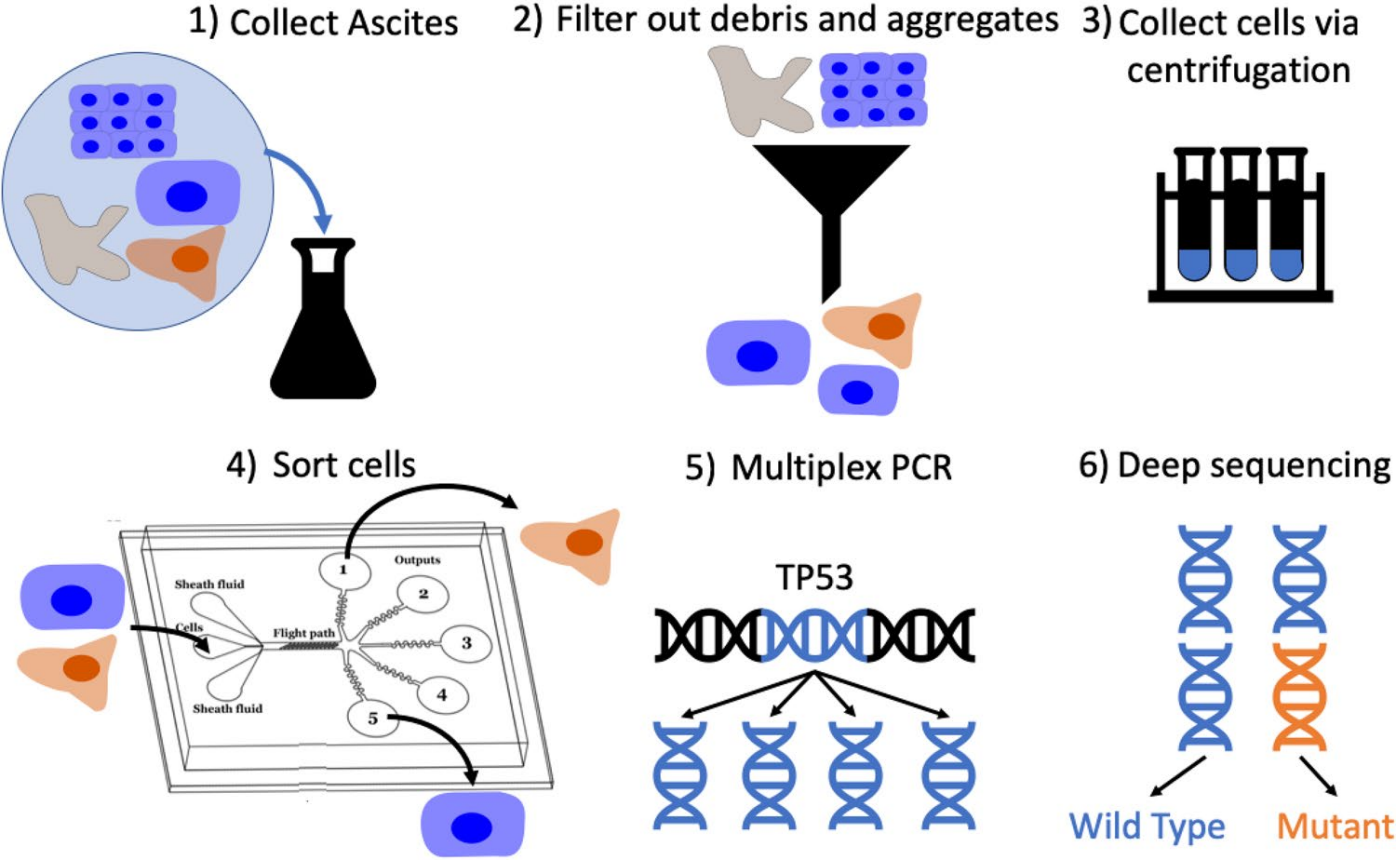
Sorting of primary ascites samples. An outline of the ascites sorting experiment: (1) Ascites containing nonmalignant cells (blue), cell aggregates, malignant cells (orange) and cell debris (grey) were collected from a patient. (2) Debris and aggregates were removed by filtration. (3) Cells were collected from the ascites via centrifugation. (4) Malignant cells were sorted from normal cells using our microfluidic platform. (5) The TP53 gene was selectively amplified from the genomic DNA isolated from each sorted fraction. (6) The proportion of cancer cells present in a sorted fraction was assessed by measuring the fraction of mutant reads present in the TP53 gene of genomic DNA isolated from each sample.

### Ascites sorting for immunocytochemistry

Primary ascites specimens were obtained from one patient from Northside Hospital (Atlanta, Georgia) under an approved Institutional Review Board (IRB H16135) (Fig. 1.1). This specimen was processed with a cell strainer (pluriSelect) to remove solid tissue and large cell aggregates greater than 20 um in size (Fig. 1.2). Cells were collected via centrifugation (Fig. 1.3) and resuspended in flow buffer (35% Percoll, 1% BSA, 1% EDTA and Tween in PBS[-,-]) and pumped through our device at a total flow rate of 27.5 μl/min (Fig. 1.4). Cells were collected from the device outlets and spun onto coverslips and fixed using 4% paraformaldehyde (PFA) for 10 min at room temperature. The fixed cells were permeabilized by incubating the cells in 0.1% Triton X-100 in PBS for 10 min at room temperature. The permeabilized cells were then incubated in blocking solution containing 1% BSA, 22.52 mg/ml glycine and 0.1% Tween-20 in PBS to block non-specific interactions. The cells were finally incubated in a 1:100 dilution of the mouse monoclonal primary antibody against transglutaminase 2 (ab2386, Abcam) for 1 h at room temperature and then incubated in a 1:200 dilution of the donkey polycolonal secondary antibody against mouse IgG (ab150105, Abcam) for 1 h at room temperature. Finally, the cells were counterstained using Hoechst. The cells were then imaged using an imaging plate reader (Biotek) and the relative brightness of each cell was quantified using the plate reader software.

## Results

**Figure 2:**
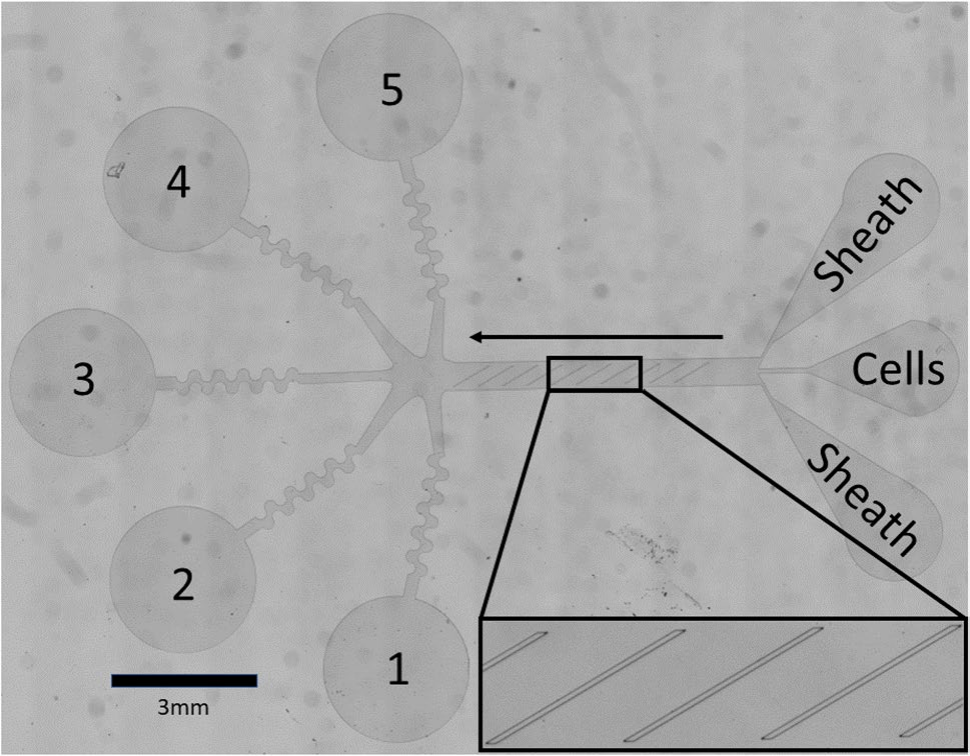
Device overview. The device consists of three inlets and five outlets connected by a sorting channel. Sheath fluid is pumped into the outer two inlets in order to focus the cells from the center inlet to their desired initial lateral position in the device, which in this application was the ‘top’ edge of the ridges (the side of the ridges closest to outlet 5). The cells then flow into the sorting chamber, where they interact with periodic diagonal constrictions (inset) which are designed to force cells to deform in order to pass under them. Large, stiff cells will tend to translate along the ridge (down in the figure, towards outlets 1–3) whereas small, soft cells will pass under the ridges without deflecting and be collected in outlets 4–5.

The purpose of this study was to develop a microfluidic device capable of sorting metastatic ovarian cancer cells from liquid patient samples by leveraging biomechanical differences between target cancer cells and contaminating nonmalignant cells. As depicted in Fig. 2, the device has 3 input ports, one for cells flanked by two for sheath liquid, which are used to organize the cells into a narrow stream aimed at the ‘top’ edge of the ridges. After entering the device, cells travel through a rectangular microchannel containing periodic diagonal constrictions. Larger, stiffer cells tend to translate along the constrictions (down in Fig. 2) towards outlets 1–3, whereas smaller, softer cells simply pass through to outlets 4–5. Therefore, cells with different biomechanical properties are directed towards different outlets, of which there are 5 in total. Performance of this device for a given application depends on careful selection of device geometry and flow rate in order to find a productive balance between hydrodynamic and mechanical forces operating on the cell. Generally, the mechanical contribution to cell trajectories can be increased by decreasing the size of the gap between the bottom of the ridges and the floor of the channel whereas the hydrodynamic contribution to cell trajectories can be increased by increasing the flow rate. When hydrodynamic forces dominate, either by selecting too large of a gap size or too high of a flow rate the cells will simply follow the streamlines in the device and no sorting will occur. If mechanical forces dominate, either through selection of too small of a gap size or too low of a flow rate cells will not be able to transit the constrictions and clogs will occur that prevent successful operation of the device.

**Figure 3:**
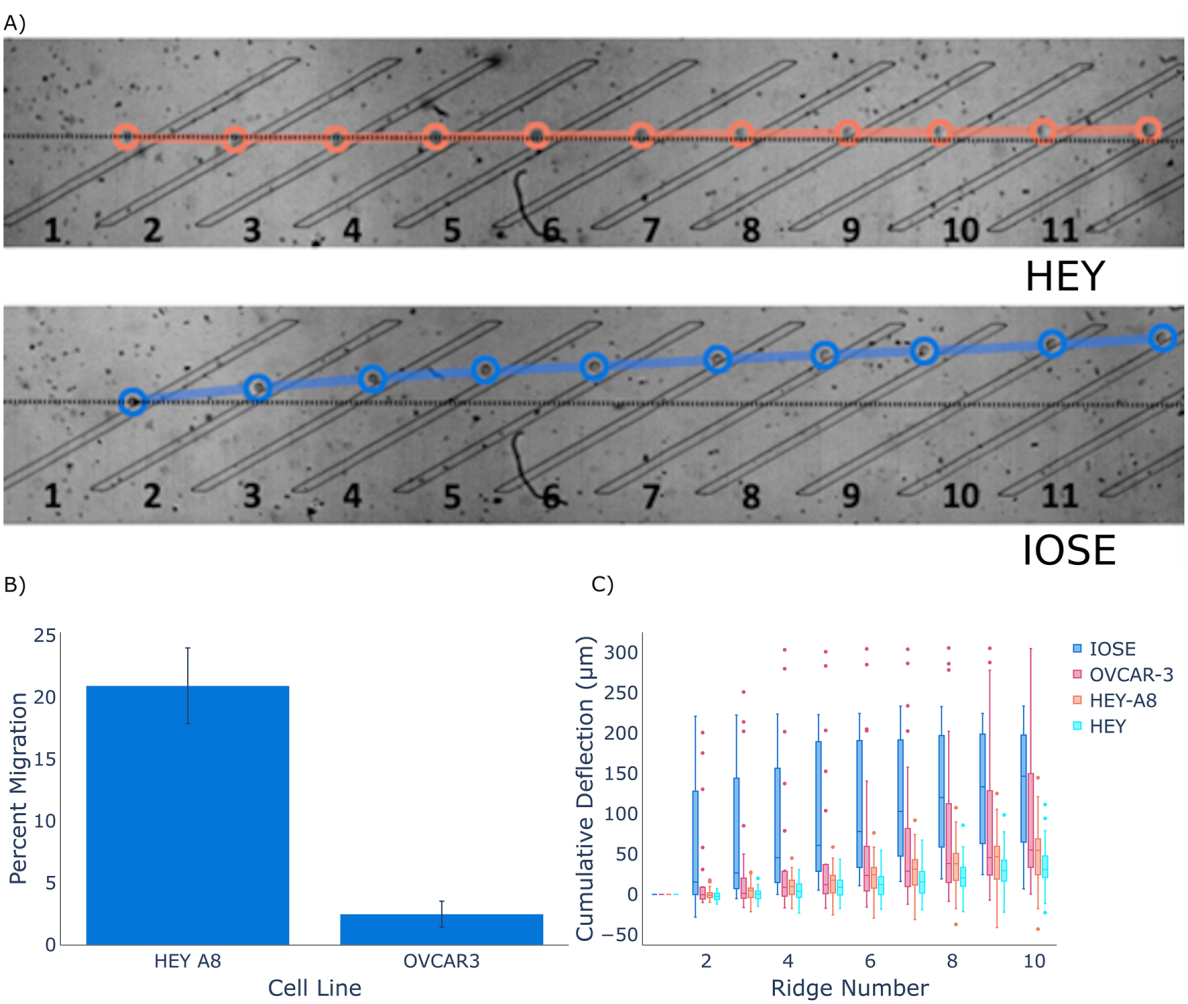
Trajectory analysis of ovarian cancer cell lines. (**A**) Characteristic trajectories of a metastatic ovarian cancer cell line (HEY) versus a nonmetastatic ovarian cancer cell line (IOSE) (**B**) Migration assay showing the relative invasiveness of the HEY and OVCAR-3 cell line. (**C**) The trajectories of a variety of ovarian cancer cell lines analyzed to show the cumulative cell deflection at each ridge. Cells with a lower metastatic ability (IOSE, OVCAR3) deflected more than cells with a higher metastatic ability (HEY, HEY-A8). The videos used for this analysis are provided as Supplemental videos.

Our first objective was to optimize the sorting device to be sensitive to the mechanical differences between ovarian cells with varying malignancy. We used high speed microscopy analysis to track cell lines of varying metastatic potential. As shown in Fig. 3, at a total flow rate of 30 µl/min differences in deflection between nonmalignant (IOSE) and malignant (HEY, HEY-A8 and OVCAR-3) cell types are observed. In addition, a substantial difference in cell trajectory was observed between cell lines with low (OVCAR3) and high (HEY-A8) metastatic potential. Therefore, biomechanical sorting is sensitive to mechanical differences between ovarian cancer cells of different metastatic potential. The dynamic range of the separation was not sufficient to substantially isolate HEY and HEY-A8 cells in this configuration.

**Figure 4:**
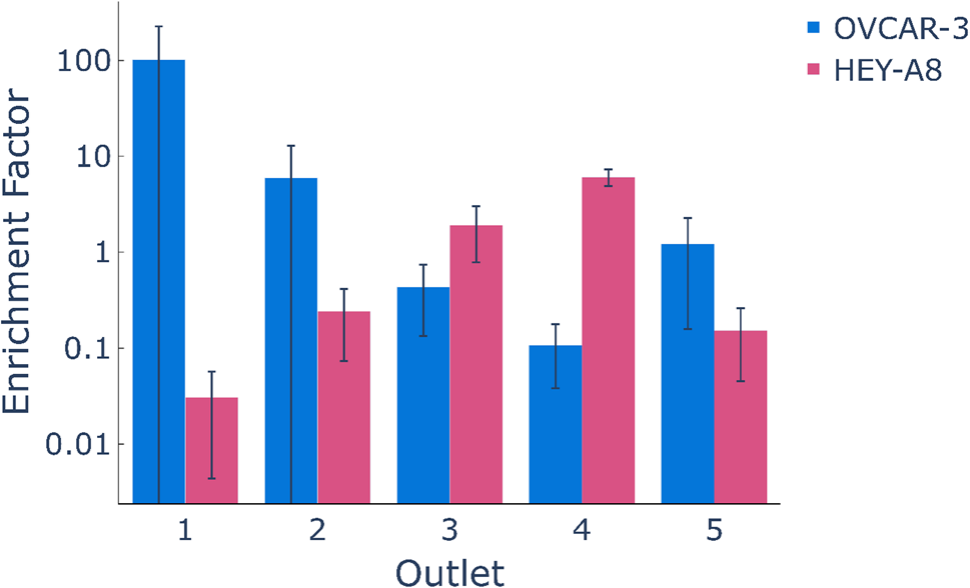
Sorting of ovarian cancer cell lines. In order to assess if our platform was capable of sorting cells with varying degrees of metastatic potential, we used our device to separate highly invasive cells (HEY-A8) from noninvasive cells (OVCAR-3). We were able to achieve enrichment factors of ~ 100-fold for OVCAR-3 and ~ sixfold for the softer HEY-A8. Outlet 5 contained relatively few cells and would not be used in a clinical application. The purity of HEY-A8 cells collected at each outlet for each trial are presented as a Supplementary table.

After finding that the cell trajectories demonstrated the device’s sensitivity to mechanical differences between ovarian cancer cell lines with differences in metastatic potential, we set out to establish that cell types of different metastatic potential can be separated. OVCAR-3 and HEY-A8 cells were labeled, mixed and infused into the device. The cells were sorted into 5 different outlets. As shown in Fig. 4, a majority of the OVCAR-3 cells translated towards the stiff outlet (Outlets 1 and 2) while the HEY A8 cells translate into soft outlets (Outlets 4 & 5). From flow cytometry analysis of the sorted subpopulations, the enrichment factor of the target cell type was calculated using the following equation:

$$\frac{{(Number\, of\, X\, cells/Number\, of\, Y\, cells)}_{Outlet}}{{(Number\, of\, X\, cells/Number\, of\, Y\, cells)}_{Inlet}}$$

As shown in Fig. 4, the enrichment factor for HEY-A8 cells increases from outlet 1 to 4 while the enrichment factor for OVCAR-3 cells decreases from outlet 1 to 4 Thus, OVCAR-3 cells are highly enriched at outlet 1 and HEY-A8 is highly enriched at outlet 4. Specifically, we were able to achieve enrichment factors of ~ 100-fold for OVCAR-3 and ~ sixfold for HEY-A8. Very few cells were sorted into outlet 5 where only the smallest and softest cells would be expected.

**Figure 5:**
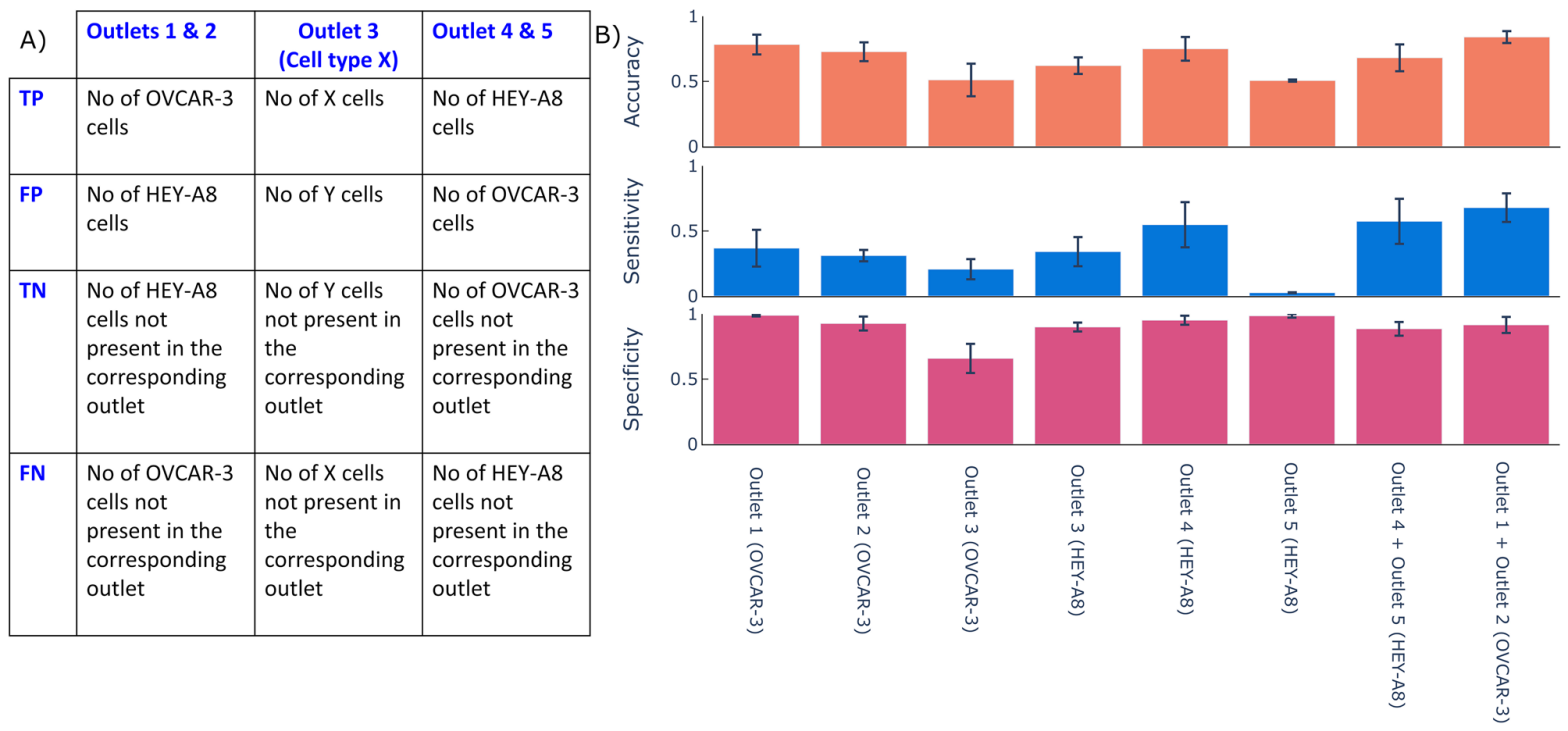
Sensitivity, specificity and accuracy analysis. (**A**) Definitions of true positive, true negative, false positive and false negative used for our sensitivity analysis. (**B**) The sensitivity, specificity and accuracy of sorting cells using our platform for each outlet, as well as for combinations of some neighboring outlets. The cell line in parentheses in the x axis label indicates which cells were considered true positives in the analysis.

A sensitivity analysis was performed on the sorted populations of metastatic HEY-A8 cells and less metastatic OVCAR-3 cells to evaluate the accuracy of biomechanical sorting. The number of cells at various outlets were divided based on the conditions as shown in the confusion matrix (Fig. 5A). Further, Fig. 5A shows the true positive (TP), false positive (FP), true negative (TN) and false negative (FN) corresponding to all five outlets, as determined from the outlet of the device (condition) and flow cytometry analyses of cell stains (test). The number of HEY-A8 cells was considered as TPs for outlets 4 and 5 while the number of OVCAR-3 cells were considered to be TPs for outlets 1 and 2.

Sensitivity is the proportion of true positives correctly sorted by the device, defined by the equation $Sensitivity=\frac{TP}{TP+FN}$. Sorting with high sensitivity indicates that most of the desired cells have been collected at a particular outlet. As shown in Fig. 4, most of the softer cells (HEY-A8) have been collected at outlets 4 or 5 and most of the stiff cells (OVCAR-3) have been collected at outlets 1 or 2.

Specificity is the proportion of true negatives correctly sorted by the device, defined by the equation $Specificity=\frac{TN}{TN+FP}$. A sorting experiment with high specificity indicates that most of the non-desired cells at the corresponding outlets have not been sorted at the outlet. For example, most of OVCAR-3 have not been collected at outlets 4 and 5, and most of HEY-A8 cells have not been collected at outlets 1 and 2. Accuracy is the proportion of true cells (TPs or TNs) in a sorted population. It indicates the degree of veracity of the sorting test, defined by the equation $Accuracy=\frac{TN+TP}{TN+TP+FN+FP}$. Figure 5B shows the calculated sensitivity, specificity and accuracy for 3 trials of cell separation using the microfluidic device and evaluating several outlet combinations. The separation with the device has a maximum sensitivity of 0.67, specificity of 0.99, and accuracy of 0.84.

**Figure 6:**
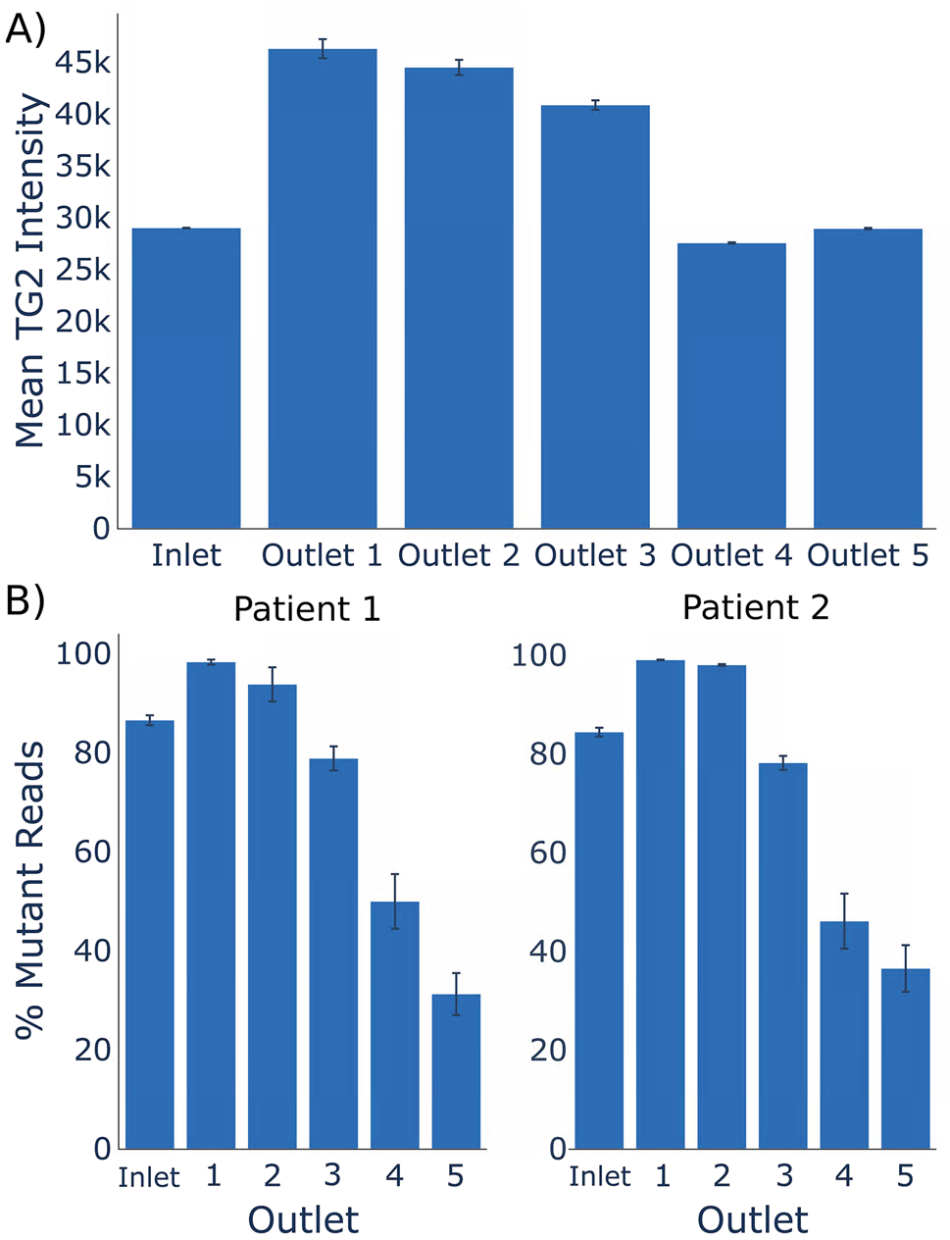
Sorting of primary ascites samples. To assess the usefulness of our platform in a clinical setting, we used our device to sort metastatic cells from patient ascites samples. Enrichment of putative cancer cells (TP53 mutant, high TG2 intensity) was achieved in outlet 1 and 2 for (**A**) one patient as quantified via immunocytology and (**B**) two separate patients as quantified via sequencing.

To determine if microfluidic sorting is capable of separating cell populations of clinical interest from heterogeneous specimens, we obtained ascites from two patients with advanced metastatic ovarian cancer from Northside Hospital in Atlanta, GA under an informed consent IRB protocol (H16135). Cells were resuspended in flow buffer and subjected to biomechanical sorting. The sorted fractions were collected from each of the 5 output ports and were stained for TG2, a protein whose overexpression has been shown to be a feature of ovarian cancer^39^. This sorting resulted in cell fractions enriched for high-TG2 cells, consistent with an enrichment of metastatic cancer cells (Fig. 6). In addition, NGS libraries were prepared to enable deep sequencing of the TP53 gene, mutations in which are commonly observed in ovarian cancer^40^. As shown in Fig. 6, the proportion of mutant reads and TG2 florescence intensity in the sorted populations change at different device outlets indicating separation of the heterogeneous input into biologically relevant subpopulations. Mutations in P53 were enriched at outlets 1 and 2 whereas in outlets 4 and 5, cells containing P53 mutations were substantially removed, indicating a transfer of cancer cells to outlets of 1 and 2. These results were consistent for both patients and indicate that the microfluidic cell sorting device is capable of selectively enriching cells with TP53 mutations associated with ovarian cancer cells in a label-free manner. A surprising feature of this result is that the cancer cells were enriched in outlets 1–2 for the primary cell sort, where metastatic cancer cells were enriched in outlets 3–4 in our cell line experiments. We believe this occurred because the cell line isolation was driven by differences in stiffness whereas the primary cell sorting was driven by differences in cell size. In the cell line experiment both cell lines were of similar size, but the cells with higher metastatic ability were softer, leading to them being sorted into outlets 3–4. In the primary cell experiment we believe sorting was driven by differences in cell size, where our cells of interest (CTCs) were larger than contaminating blood and immune cells. Therefore, our larger cells of interest deflected more than the smaller contaminating cells, resulting in enrichment of our cancer cells in outlets 1–2.

## Discussion

Personalized medicine is an exciting opportunity in cancer treatment that promises to increase the effectiveness of cancer therapy by accounting for differences between each patient's specific disease. However, application of personalized medicine depends on the clinician’s ability to identify the specific characteristics of a patient's cancer cells. The collection of a minority of tumor cells from complex liquid biopsies has been proposed as an effective yet minimally invasive approach to collecting metastatic cells from patients for testing. However, for testing to be economically feasible, technologies are required to perform high-throughput sorting of cancer cells from patient samples. Alternatively, it is possible to use high-cost next generation sequencing methods to analyze rare subsets of tumor cells, but a much higher sequencing depth, at greater cost, will be required unless an enrichment strategy is utilized. By simply examining the contents of the enriched ‘cancer’ outlet post-sort, our device can potentially serve as a diagnostic tool, as the cancer burden could be examined by the ratio of cancer to noncancer cells present in a sample. Further, functional or drug-sensitivity assays are also possible to examine isolated cells during downstream testing of pharmacological agents^41,42^. Current 'gold standard' sorting techniques rely on expensive antibody-based conjugation of fluorescent markers or magnetic beads to the cells. Antibody-based strategies may affect the behavior of cells in downstream testing, whereas label-free approaches cause relatively little change to sorted cells. In addition, current gold standard technologies are particularly difficult to apply to the problem of ovarian cancer due to the lack of specific extracellular markers.

In this work, we have demonstrated a label-free, high throughput sorting platform for the isolation of malignant cells from primary ascites samples. We have shown that our device is sensitive to ovarian cancer cell lines with various degrees of metastatic potential and performed extensive characterization of our ability to sort these cell lines. Finally, we have demonstrated our ability to specifically enrich metastatic cancer cells from liquid patient samples, both by staining for the protein marker TG2 and by quantifying mutations in the cancer repressor gene TP53. Our platform constitutes an enabling step for the testing of metastatic cells for personalized medicine applications. By cheaply and quickly isolating metastatic cells from complex patient samples, we will enable downstream assays for drug response and functional assays as well as point-of-care diagnostics.

## Supplementary Information

Supplementary Information 1.

Supplementary Information 2.

Supplementary Information 3.

Supplementary Information 4.

Supplementary Information 5.

Supplementary Information 6.
